# Normative models for neuroimaging markers: Impact of model selection, sample size and evaluation criteria

**DOI:** 10.1016/j.neuroimage.2023.119864

**Published:** 2023-03

**Authors:** Jelena Bozek, Ludovica Griffanti, Stephan Lau, Mark Jenkinson

**Affiliations:** a Faculty of Electrical Engineering and Computing, University of Zagreb, Zagreb, Croatia; b Wellcome Centre for Integrative Neuroimaging, Oxford Centre for Human Brain Activity, Department of Psychiatry, Warneford Hospital, University of Oxford, United Kingdom; c Wellcome Centre for Integrative Neuroimaging, Centre for Functional MRI of the Brain (FMRIB), Nuffield Department of Clinical Neurosciences, John Radcliffe Hospital, University of Oxford, United Kingdom; d Australian Institute for Machine Learning, School of Computer and Mathematical Sciences, The University of Adelaide, Adelaide, SA, Australia; e South Australian Health and Medical Research Institute (SAHMRI), Adelaide, SA, Australia

**Keywords:** Normative modelling, MRI, GAMLSS, Big data, Brain ageing

## Abstract

Modelling population reference curves or normative modelling is increasingly used with the advent of large neuroimaging studies. In this paper we assess the performance of fitting methods from the perspective of clinical applications and investigate the influence of the sample size. Further, we evaluate linear and non-linear models for percentile curve estimation and highlight how the bias-variance trade-off manifests in typical neuroimaging data.

We created plausible ground truth distributions of hippocampal volumes in the age range of 45 to 80 years, as an example application. Based on these distributions we repeatedly simulated samples for sizes between 50 and 50,000 data points, and for each simulated sample we fitted a range of normative models. We compared the fitted models and their variability across repetitions to the ground truth, with specific focus on the outer percentiles (1st, 5th, 10th) as these are the most clinically relevant.

Our results quantify the expected decreasing trend in variance of the volume estimates with increasing sample size. However, bias in the volume estimates only decreases a modest amount, without much improvement at large sample sizes. The uncertainty of model performance is substantial for what would often be considered large samples in a neuroimaging context and rises dramatically at the ends of the age range, where fewer data points exist. Flexible models perform better across sample sizes, especially for non-linear ground truth.

Surprisingly large samples of several thousand data points are needed to accurately capture outlying percentiles across the age range for applications in research and clinical settings. Performance evaluation methods should assess both bias and variance. Furthermore, caution is needed when attempting to go near the ends of the age range captured by the source data set and, as is a well known general principle, extrapolation beyond the age range should always be avoided. To help with such evaluations of normative models we have made our code available to guide researchers developing or utilising normative models.

## Highlights

• We propose a simulation-based assessment of variance and bias of normative models.
• We investigate the impact of sample size, ground truth, fitting model and percentile.
• Precise estimation of outlying percentiles requires large samples (e.g. N≫1000).
• Uncertainty rises greatly at ends of the age range, where fewer data points exist.
• We provide an open tool that can be used for the equivalent of power calculations.

## 1 Introduction

Normative models are a useful tool for predicting and assessing measures for an individual relative to the population. They provide an estimate of all or part of a conditional probability distribution (often conditioned on age) for a reference population of ‘normal’ (often ‘healthy’) individuals, allowing quantification of how a certain individual might deviate from this reference population. While they are well established in some clinical contexts (e.g. paediatric growth charts (Borghi, de Onis, Garza, Van den Broeck, Frongillo, Grummer-Strawn, Van Buuren, Pan, Molinari, Martorell, Onyango, Martines, for the WHO Multicentre Growth Reference Study Group, 2006, WHO Multicentre Growth Reference Study Group, de Onis, 2006)), neuroimaging has started to adopt them only recently, where they have already been beneficial for the assessment of brain development (Chen, Holmes, Zuo, Dong, 2021, Dimitrova, Arulkumaran, Carney, Chew, Falconer, Ciarrusta, Wolfers, Batalle, Cordero-Grande, Price, Teixeira, Hughes, Egloff, Hutter, Makropoulos, Robinson, Schuh, Vecchiato, Steinweg, Macleod, Marquand, McAlonan, Rutherford, Counsell, Smith, Rueckert, Hajnal, O’Muircheartaigh, Edwards, 2021, Erus, Battapady, Satterthwaite, Hakonarson, Gur, Davatzikos, Gur, 2015), ageing (Bethlehem, Seidlitz, White, Vogel, et al., 2022, Rutherford, Fraza, Dinga, Kia, Wolfers, Zabihi, Berthet, Worker, Verdi, Andrews, Han, Bayer, Dazzan, McGuire, Mocking, Schene, Sripada, Tso, Duval, Chang, Penninx, Heitzeg, Burt, Hyde, Amaral, Wu Nordahl, Andreasssen, Westlye, Zahn, Ruhe, Beckmann, Marquand, 2022) and in various clinical conditions related to psychiatry (Marquand, Kia, Zabihi, Wolfers, Buitelaar, Beckmann, 2019, Wolfers, Beckmann, Hoogman, Buitelaar, Franke, Marquand, 2020, Wolfers, Doan, Kaufmann, Alnæs, Moberget, Agartz, Buitelaar, Ueland, Melle, Franke, Andreassen, Beckmann, Westlye, Marquand, 2018, Zabihi, Oldehinkel, Wolfers, Frouin, Goyard, Loth, Charman, Tillmann, Banaschewski, Dumas, Holt, Baron-Cohen, Durston, Bölte, Murphy, Ecker, Buitelaar, Beckmann, Marquand, 2019) and dementia (Pinaya et al., 2021). Moving away from group-level studies, normative modelling incorporates heterogeneity in clinical cohorts, allowing predictions at an individual participant level (Marquand et al., 2016).

The long-term perspective is to develop effective quantitative neuroradiology tools to support clinical decision making. The Quantitative Neuroradiology Initiative (QNI) framework (Goodkin et al., 2019) provides practical steps, which emphasise compiling reference data to contextualise patient’s findings. An example of a promising application of normative modelling of brain measures in clinical practice is in the area of dementia diagnosis (Goodkin et al., 2019). In particular, hippocampal atrophy has been included in the diagnostic criteria for Alzheimer’s disease (AD) since 2011 (McKhann et al., 2011). Clinicians perceive information on the hippocampal volume as a valuable biomarker for cognitive impairment evaluation in suspected Alzheimer’s disease patients (Bosco et al., 2017) and a European survey conducted in 2019 (Vernooij et al., 2019) reports that, among the centres that use quantitative evaluations, hippocampal volume is the most frequently measured (with freely available software or commercial products). Thus, computation of normative curves for hippocampal volume for an early assessment of disease onset has the potential to provide valuable and more rigorously quantitative assistance to clinicians.

The current state is that studies collect huge amounts of data, such as ADNI (Jack et al., 2008), ABIDE (Martino et al., 2014), dHCP (Hughes, Winchman, Padormo, Teixeira, Wurie, Sharma, Fox, Hutter, Cordero-Grande, Price, Allsop, Bueno-Conde, Tusor, Arichi, Edwards, Rutherford, Counsell, Hajnal, 2017, Makropoulos, Robinson, Schuh, Wright, Fitzgibbon, Bozek, Counsell, Steinweg, Vecchiato, Passerat-Palmbach, Lenz, Mortari, Tenev, Duff, Bastiani, Cordero-Grande, Hughes, Tusor, Tournier, Hutter, Price, Teixeira, Murgasova, Victor, Kelly, Rutherford, Smith, Edwards, Hajnal, Jenkinson, Rueckert, 2018), HCP (Van Essen et al., 2013), UK Biobank (UKB) (Miller et al., 2016). This makes it possible to conduct big population studies and apply normative models to brain MR imaging data. Generally, having a large sample size allows more precise normative distributions to be calculated. Sample size calculations have been performed in other areas like paediatric growth charts, with a recent study concluding that 7000–25,000 participants per sex are needed to model growth from 0 to 21 years (Cole, 2021). However, it is not known what is the minimal sample size for providing a realistic and sufficiently accurate normative model for neuroimaging applications. This is important as even though neuroimaging data sets are now much larger than before, many studies derive normative distributions from much smaller data sets, with as little as a few hundred participants (Ber, Hoffman, Hoffman, Polat, Derazne, Mayer, Katorza, 2017, Dong, Castellanos, Yang, Zhang, Zhou, He, Zhang, Xu, Holmes, Thomas Yeo, Chen, Wang, Beckmann, White, Sporns, Qiu, Feng, Chen, Liu, Chen, Weng, Milham, Zuo, 2020, Lv, Di Biase, Cash, Cocchi, Cropley, Klauser, Tian, Bayer, Schmaal, Cetin-Karayumak, et al., 2021, Schmidt-Richberg, Ledig, Guerrero, Molina-Abril, Frangi, Rueckerton behalf of the Alzheimer’s Disease Neuroimaging Initiative, 2016, Vinke, Huizinga, Bergtholdt, Adams, Steketee, Papma, de Jong, Niessen, Ikram, Wenzel, Vernooij, 2019).

The development and choice of the best method to generate a normative model for a particular application is crucial and a very active area of research. Normative modelling methods that are currently available use a number of different techniques, such as hierarchical linear models, polynomial or quantile regressors, Gaussian process models and support vector machines (Marquand et al., 2019). However, each technique has some weaknesses, for example, with Gaussian process regression it is hard to accurately estimate the aleatoric uncertainty with sparse data (Xu et al., 2021), linear models do not capture non-linear relationships, and other methods make assumptions of Gaussianity about the conditional distribution (i.e. a consistent and symmetric relationship between all the percentile curves). One good candidate for more flexible modelling is the generalized additive model, often implemented using the VGAM (Vector Generalized Linear and Additive Models) (Yee, 2015) or GAMLSS (Generalized Additive Models for Location Scale and Shape) (Rigby and Stasinopoulos, 2005) packages.

GAMLSS is a very flexible unifying framework for univariate regression (Stasinopoulos et al., 2017) that accommodates a wide range of distribution models where all the parameters of the distribution can be modelled as a function of the explanatory variables. It therefore extends basic statistical models, allowing flexible modelling of non-constant variance, skewness and kurtosis in the data.

There are many examples of GAMLSS being used in practice for normative modelling in neuroimaging, with a range of training sample sizes; for example, 94 foetal images in Ber et al. (2017), 948 paediatric images in Dong et al. (2020), 19,793 adult images in Nobis et al. (2019), 25,575 paediatric and adult images in Córdova-Palomera et al. (2021), and up to 123,984 images across the majority of the lifespan in Bethlehem et al. (2022). This demonstrates the wide range of training sizes used, but despite this the issue of whether the number of images used for training is sufficiently accurate, and quantitative measures of accuracy, are rarely discussed.

Alternative methods to GAMLSS have also been used in neuroimaging and include the lambda-mu-sigma (LMS) method and implementations in VGAM (Schmidt-Richberg, Ledig, Guerrero, Molina-Abril, Frangi, Rueckerton behalf of the Alzheimer’s Disease Neuroimaging Initiative, 2016, Vernooij, Jasperse, Steketee, Koek, Vrooman, Ikram, Papma, van der Lugt, Smits, Niessen, 2018, Vinke, Huizinga, Bergtholdt, Adams, Steketee, Papma, de Jong, Niessen, Ikram, Wenzel, Vernooij, 2019) (with training sizes of 248 through to 4915 in these papers), warped Bayesian linear regression (Fraza et al., 2021) (with 20,083 adult images), sliding window approaches with both fixed and variable window sizes, such as Nobis et al. (2019) (with 19,793 adult images) and Janahi et al. (2022) (with 40,000 adult images), as well as Gaussian Process Regression (Janahi et al., 2022).

A common factor across many implementations is the use of a transformation function, such as affine, Box-Cox and Sinh-Arcsinh (SHASH), applied to a standard normal distribution. Two notable works that have investigated the merits of different transformations (Dinga, Fraza, Bayer, Kia, Beckmann, Marquand, Fraza, Dinga, Beckmann, Marquand, 2021) both concluded that SHASH was the best transformation, with the latter paper using GAMLSS. We will therefore show many results from GAMLSS with SHASH in our results, although we tested several alternatives as well.

Evaluation of the normative model obtained in most studies in neuroimaging to date is either missing or not performed in ways that address the key needs of the intended application. For example, in clinical settings it is common to estimate the outer percentile curves (e.g., the 5th percentile), to identify participants that are most likely to have some disease. Assessing errors in the central tendency or explained variance (Rutherford et al., 2022b) does not provide crucial information about the accuracy of key percentile estimates. Furthermore, providing breakdowns of errors so that the errors at the ends of the distribution can be assessed is important given that, in real life applications, the density of data points typically decreases at one or both ends of the age range in the sample. Knowing the behaviour at the edges is important for setting the practical limits of the normative model and estimating the likely performance of any extrapolation. Another factor that needs to be considered in evaluation is whether bias can be assessed, which is difficult to accurately do without ground truth or massive amounts of data.

Statistical uncertainties exist whenever an estimation method is used and a normative model is no different, although it is less intuitive to grasp what an uncertainty on a probability value means, especially for non-technical end users such as clinicians and patients. However, uncertainties can be presented in user-friendly terms for clinical use cases where the likelihood of being below a critical percentile (e.g. the 1% level) is shown as something like a colour-coded result – for example, red to green where red is highly likely to be under 1% (and potentially abnormal) and green is very unlikely to be under 1%. For technical end users, such as neuroimaging researchers, the uncertainty can be incorporated into statistical models to provide confidence intervals of percentiles or used as part of the inputs to statistical tests used to assess the differences between groups and/or individuals.

In this study we evaluate the effect of sample size and model selection on normative models for neuroimaging markers, using hippocampal volume as an exemplar to help with the narrative but without this in any way limiting the scope of our investigations. Our approach is to use a range of simulated data from a known ground truth and we can therefore assess bias and variance in any of the percentile curves. From this we aim to provide guidelines on choosing appropriate sample sizes and fitting methods with respect to the levels of bias and variance that can be expected. The investigations focus on the outer percentiles (1st, 5th and 10th percentiles), as these are the most clinically relevant. Given our example application to hippocampal volume, these percentiles would be useful for detecting hippocampal atrophy. Janahi et al. (2022) recently showed that AD patients from the ADNI data set fall on average around the 1st percentile derived from UK Biobank data, with most of them falling below the 2.5th percentile. They also showed that people with mild cognitive impairment who then progressed to AD had an average hippocampal volume percentile of 11%. Commercial products reporting various metrics (including hippocampal volumes) against normative data use different thresholds to flag abnormal cases, but all between the 1st and 5th percentile (e.g. Icometrix® (Icometrix NV, 2022), Brainminer (Brainminer Ltd., 2022), NeuroQuant (Cortechs.ai Inc., 2022)). For neuroimaging measures where abnormal cases would be represented by higher values (e.g. white matter hyperintensities), a similar approach to what we present here would be applicable to the 90th, 95th and 99th percentiles.

A range of models are included, but our goal is not to perform an exhaustive search to find the best possible model, and so a number of commonly applied modelling methods are included (e.g. GAMLSS and sliding windows), which are intended to be representative of the range of methods used in practice. Our approach can easily be used to evaluate the performance of any normative model. It could therefore be used as a power calculation tool to assess the expected variance and bias of the percentiles of interest for particular normative modelling studies and applications.

## 2 Methods

**Fig. 1:**
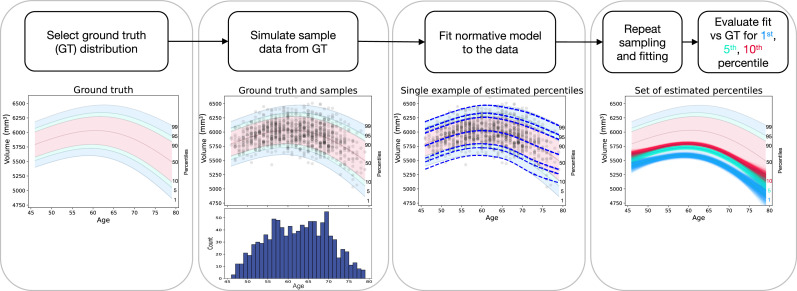
Overview of the framework for simulating and fitting a normative modelling method. The diagram summarises the methodological steps performed in the present study. For each step, an example result is given for a simulated sample size of $N_{s}=1000$ and one modelling method. For more details on the different options tested for each step, please refer to the main text.

An overview of the workflow for one of the normative modelling methods is presented in Fig. 1. We initially select an analytical ground truth distribution for a scalar quantity (what we will refer to as hippocampal volumes as an exemplar) in the age range of 45 to 80 years (to approximately match the UK Biobank study). This distribution is then used to create many simulated samples and the normative modelling method is to fit to each simulated sample. Multiple sets of simulated data were generated for each sample size (where sample sizes vary from 50 to 50,000) so that for each model, at each sample size, there were many fits. Finally, we evaluated how close the fitted percentile curves are to the ground truth curves, concentrating on the 1st, 5th and 10th percentiles as these have the most clinical utility for the hippocampal exemplar that we are using (i.e. low hippocampal volume being indicative of hippocampal atrophy).

### 2.1 Simulated samples

We generated samples with the following sizes: 50, 100, 200, 500, 1000, 2000, 5000 and 50,000, each data point/participant corresponding to one scalar value (hippocampal volume) within an age range 45 to 80 years. For each sample size, $N_{s}$, we randomly generated $N_{d}$ simulated samples (i.e. a set of hippocampal volumes) where $N_{d}$ was 1000 except for $N_{s}=50,000$. In that case we set $N_{d}$ to 100 since, unsurprisingly, the variation in the results was a lot less and we considered that the additional data transfer and computational times were not justified.

Two ground truth distributions were chosen to cover the simplest possible case and a slightly more difficult, but plausible, case: (i) linear mean and constant variance (LinMean_ConstVar), and (ii) non-linear mean and non-constant variance (NonLinMean_NonConstVar). More precisely, the two ground truth functions were:

(1) $$\begin{array}{cc} \left(i)\;\mu (x\right) & =5000-7x,\\ \;\;\sigma (x) & =300, \end{array}$$

(2) $$\begin{array}{cc} \left(ii)\;\mu (x\right) & =-70\;(x-65)\;s((x-65)/10)+20(x-65)+6000,\\ \;\;\sigma (x) & =5\;(x-65)\;s((x-65)/10)+(x-65)+200; \end{array}$$

where x is age in years and s(x)=1/(1+exp(−x)) is the sigmoid function. In the latter case μ(65)=6000,σ(65)=200 and because the sigmoid is near zero for large negative values and near one for large positive values, it smoothly interpolates between the asymptotic lines μ(x)=20(x−65)+6000 and μ(x)=−50(x−65)+6000, where the scaling of (x−65) inside the sigmoid controls how rapid the transition is between these.

In both cases the conditional distribution, p(y|x) (i.e. for hippocampal volume *y* at a fixed age *x*) is Gaussian, with mean of μ and standard deviation of σ. Non-Gaussian distributions could easily be incorporated into the simulations, but we were more interested in how well the normative models would perform in relatively simple circumstances, given that we do not know how non-Gaussian something like hippocampal volume is likely to be in reality.

Since our aim was to build a ground truth distribution that was generally applicable to a wide range of neuroimaging measures, not one restricted to model hippocampal volume only, the equations are not strictly derived from real-life hippocampal studies, but designed to be biologically plausible in general. Example values of total hippocampal volumes reported in the literature vary from approximately 5000 mm${}^{3}$ in ADNI (e.g. 5200 mm${}^{3}$ in 200 controls in Leung et al. (2010) with a standard deviation of approx. 12%) to 7000–8000 mm${}^{3}$ in the UK Biobank (e.g. 7700 mm${}^{3}$ in 19,793 controls in Nobis et al. (2019) with a 10% standard deviation). The NonLinMean_NonConstVar case that we use here has a peak around 63 years, much like the trajectory of hippocampal volumes in Nobis et al. (2019) that have a similar inflection point. It is also worth noting that the vast majority of normative modelling methods will shift and scale outputs in exact correspondence to any additive offsets and linear scaling applied to the input data, which means that the absolute values of the mean value and scaling of the functions used will have no effect on any relative measures of performance.

The distribution of ages was not uniform but chosen to be approximately similar to the UK Biobank project (Miller et al., 2016) distribution, as this shows the typical characteristic of having fewer participants at the ends of the age range (see one example at the bottom of the second panel in Fig. 1 and supplementary Fig. S1 for other sample sizes). For each sample size a set of ages was chosen randomly from this distribution but then fixed for all simulated samples with that size, so that the values of interest (e.g. hippocampal volumes) were varied but the participant ages were not, to limit the variation being tested to one variable only. This is again a slightly simplified setting, as if both were to vary then the results obtained would likely be even more variable.

### 2.2 Normative models and fitting methods

We used fitting methods based on sliding windows and generalized additive models for location, scale and shape (GAMLSS).

A sliding window method is a model-free analysis in which an age-window of a variable or fixed size is moved along the age axis, calculating a summary quantity (e.g. average or percentile) of all values falling within the window. We have implemented two versions: (i) a fixed age window of size 5 years (SliWinW5); and (ii) a variable window where the size is adjusted to include 10% of the participants for each centre position (SliWinP10), which matches Nobis et al. (2019). The latter case has a potential advantage at the lower and higher ends of the age range where the number of participants is sparser, as this would then adjust the size to include a wider age range, although it also might over-regularise the curves by doing this. In both cases the result was then slightly smoothed using a Gaussian kernel with full width at half maximum (FWHM) of 5 years.

GAMLSS is, in general, a four-parameter distribution, modelling μ, σ, ν and τ, which are shape parameters of the distribution related to the mean, variance, skewness and kurtosis of the distribution. The implementation we used for the gamlss() function came from package gamlss (version 5.3–4) (Stasinopoulos et al., 2017). We used several GAMLSS models, implementing different methods of representing changes of the parameters with age, such as linear fitting or cubic spline smoothing across age, together with a Box Cox T (BCT) (Rigby and Stasinopoulos, 2006) or a SinhArcsinh (SHASH) (Jones, 2005) transformation, both of which create four parameter continuous distributions.

More specifically, we used the following models: (i) linear fitting (denoted as BCT-linear or SHASH-linear); (ii) cubic spline smoothing where only one parameter out of four, μ, was modelled as a function of age (denoted as BCT-μ and SHASH-μ); and (iii) cubic spline smoothing where two parameters out of four were modelled as a function of age, namely location μ and scale σ, (denoted as BCT-μ−σ and SHASH-μ−σ). The remaining parameters (including skewness and kurtosis) were kept constant with respect to age but were estimated from the data and not dependent on other variables, following the conclusions of Dinga et al. (2021). This simpler model, which varies two parameters but keeps the other two fixed with values that allow non-Gaussian distributions to be represented, showed minimal differences between the predictions compared to the version that allowed all four parameters to depend on age, in the neuroimaging-based experiments performed by Dinga and colleagues. Smoothing parameters were set to the default values provided by GAMLSS (using cs() function with 3 degrees of freedom (df)). For further details see Stasinopoulos et al. (2017).

### 2.3 Evaluation

As the simulations are based on known ground truth, both bias and variance can be assessed. This was one of the main reasons for conducting this study. We were also primarily interested in the ability to model the outer, clinically-relevant percentiles. Consequently, we used two main types of evaluation: (i) comparisons of the model percentile curves with the ground truth curves in terms of difference in the principal quantity of interest (e.g. hippocampal volume), and (ii) calculation of the percentage of the ground truth distribution falling beneath a model’s estimated percentile curve (i.e. what the true percentile is for each point on the estimated curve). In each case we assessed both the bias and variance by using signed errors, in volume or percentile values. Central values (median, mean, etc.) measure the bias (i.e. consistent offsets from the true value) and the width of the distribution (interquartile range (IQR), standard deviation, etc.) measures the variance or variability in the estimates.

In common practical settings the variance can be measured easily (e.g., measuring variation in cross validation methods across different folds) but it is much more difficult to assess bias without knowing the ground truth. The percentage of test set data points beneath a model curve can be estimated from real data when the ground truth is not known, but this requires extremely large samples for accurate estimation.

For example, using the binomial distribution for binned data points would give a standard deviation for a *p*-value estimate (i.e., a percentile) of $\sqrt{p(1-p)/N}$ such that for p=0.01 this requires $N_{s}=396$ (within a single bin) to obtain an estimate of 0.01±0.005, or $N_{s}=2475$ to reduce it to 0.01±0.002. More sophisticated evaluation estimation methods can improve a little on this, but it clearly shows the order of magnitude required, which demonstrates that extremely large test set sizes are required for accurate evaluations.

The ground truth distribution is defined by the conditional probability density g(y|x), where y is the hippocampal volume and x is the age. From this the associated cumulative distribution (along y) can be defined as $G\left(y|x\right)=\int_{-\infty }^{y}g\left(y^{\prime }|x\right)dy^{\prime }$. A percentile curve, for a fixed percentile value p, is then implicitly defined as the points where G(y|x)=p; i.e., a curve with respect to x, for fixed p, given by $y_{g}\left(x,p\right)$ where $G(y_{g}\left(x,p\right)|x)=p$.

When a particular sample of size $N_{s}$ has been fit by a normative model, it either explicitly or implicitly defines an estimated conditional density f(y|x), along with the associated percentile curves $y_{f}\left(x,p\right)$.

The two performance measures that we will use are:

(3) $$E_{1,p}\left(x\right)=\Delta y=y_{f}\left(x,p\right)-y_{g}\left(x,p\right)$$

(4) $$E_{2,p}\left(x\right)=\Delta p=G(y_{f}\left(x,p\right)|x)-p$$

where $E_{1,p}$ is the difference between the estimated and ground truth percentile curves, in units of hippocampal volume, and $E_{2,p}$ is the difference between the true percentile, p and the percentile at the estimated value $y_{f}\left(x;p\right)$, where G(…) maps the volume value to the cumulative probability (i.e. the percentage of the ground truth density under this value).

When summarising the distribution of these error values over instances of simulated data we use: (i) 95% range of volumes for $E_{1}\left(x\right)$ (calculated as the 97.5th percentile - 2.5th percentile of the $E_{1}\left(x\right)$ values across simulations); and (ii) mean absolute error (MAE) for $E_{2}\left(x\right)$ (i.e. mean($|E_{2}\left(x\right)|$)). When values are summarised over ages, the value at each age is counted equally (regardless of how many data points exist with that age) for calculating the mean. In this way the average is not dominated by the errors in the central portion of the data set, where the errors are often lower. Note that we never summarise results across different percentiles, and whenever the p index is missing on the error (e.g. $E_{1}$ and $E_{2}$) it should be considered to be there implicitly.

### 2.4 Summary

The different options being explored here are outlined in Table 1. This shows that there are 5 different options, with anywhere from 2 to 8 possible settings, leading to a large number of different combinations to explore. Results for all combinations were generated, but these will be presented in a systematic way, keeping certain options fixed or summarising over the different settings, so that the most important aspects are clearly laid out.

**Table 1:** Summary of the different options explored in this work.

Option	Possible settings
Sample Size, $N_{s}$	50, 100, 500, 1000, 2000, 5000, 50,000
Ground Truth	Linear-Constant-Variance
	Non-linear-Non-Constant-Variance
Estimated Percentile, p	1, 5, 10
Fitting Methods	Fixed-Width Sliding Window (5 years)
	Variable Sliding Window (10% of data points)
	GAMLSS with BCT or SHASH distribution
	... combined with linear, μ or μ−σ models
Error Measure	$E_{1}$ for volume
	$E_{2}$ for percentage of data points under the curve

## 3 Results

The following sections will present results dissected in different ways, starting by looking at the effects of sample size and fitting method in general. Following this is a more detailed look at how the results vary with age, across different percentiles and sample sizes. The last result then more explicitly investigates the bias and variance components, with particular emphasis on their relation with age and sample size.

### 3.1 Sample size

**Fig. 2:**
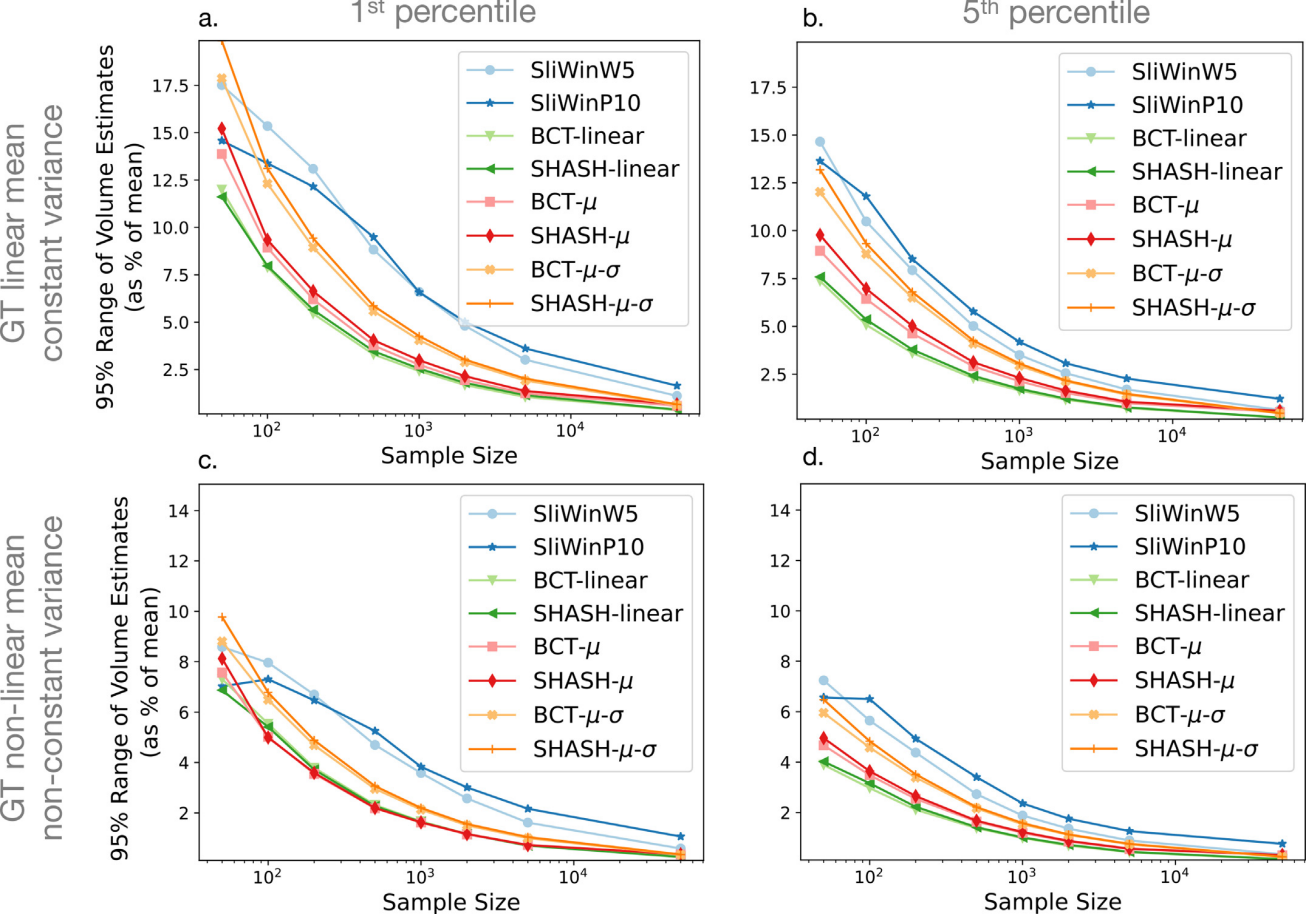
95% range of volume estimates, $E_{1}$, against sample size for each fitting method. The plots show the mean across age of the 95% intervals of volume errors ($E_{1}$), shown as a percentage of the mean, of the 1st percentile (left column) and 5th percentile (right column) curves, for linear mean and constant variance ground truth (top row) and non-linear mean and non-constant variance ground truth (bottom row). Results for the 10th percentile were very similar to those for the 5th percentile and are shown in Supplementary Fig. S2.

Figure 2 shows the effect of sample size on the errors in the volume estimates, $E_{1}$. The 95% interval is used to purely capture the variance of the error, independent of bias, and is shown for the 1st and 5th percentiles (columns) with different ground truth functions (rows). The values are calculated for each age, across all the simulated samples at a given sample size, and then the mean across the age range is taken, equally weighting each age. Results for 10th percentile are similar and can be found in the supplementary material.

**Fig. 3:**
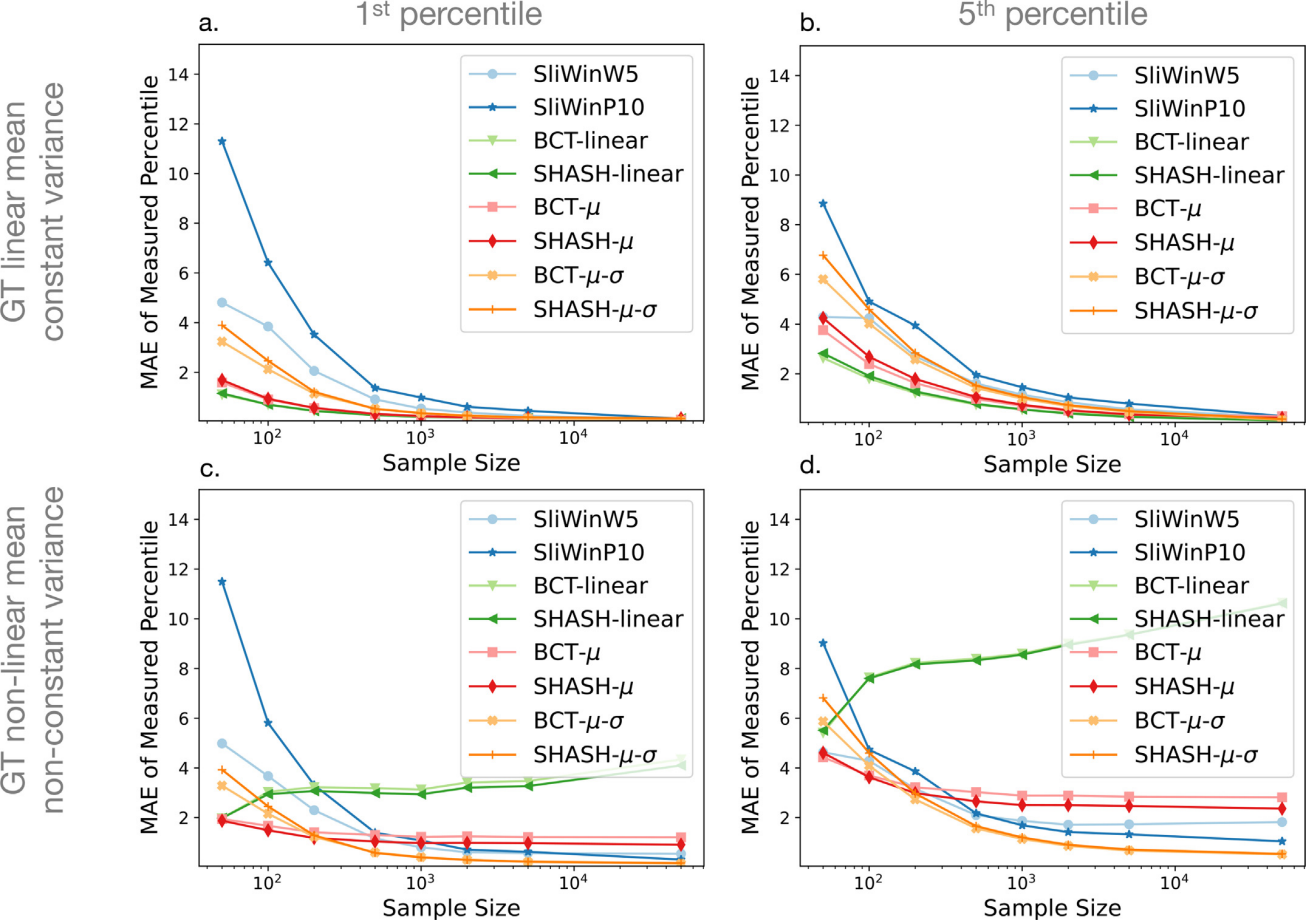
Mean Absolute Error (MAE - dimensionless) of $E_{2}$ against sample size for each fitting method. The plots show the mean across age of the mean absolute error of the estimates of percentile error ($E_{2}$) of the 1st percentile (left column) and 5th percentile (right column) curves for linear mean and constant variance ground truth (top row) and non-linear mean and non-constant variance ground truth (bottom row). Results for the 10th percentile were very similar to those for the 5th percentile and are shown in Supplementary Fig. S3.

Similarly, Fig. 3 shows the effect of sample size on the errors in percentile value, $E_{2}$. The mean absolute error (MAE) in this case reflects both bias and variance in the error, and is used partly because of the floor effect in $E_{2}$, as a percentile cannot be less than zero. The same type of limit does not apply in the other direction. e.g. errors can easily be 5% or more on one side. Note that in the tail of the distribution the difference in behaviour between the two error estimates, $E_{1}$ and $E_{2}$, becomes greater due to the fact that the percentile curves are spaced unevenly, becoming more widely spaced in the tails (very low or very high percentiles). For example, in a Gaussian distribution with μ=6000 and σ=200 (our non-linear case for an age of 65 years) the difference in volumes between the 1st and 2^nd^ percentiles is 54.5 mm${}^{3}$ whereas between the 5th and 6th percentiles it is 18.0 mm${}^{3}$. Consequently, a change of 0.5% would equate to a volume change of 9 mm${}^{3}$ if it is around the 5th percentile but would equate to three times as much volume change (over 27 mm${}^{3}$) if it is around the 1st percentile. Therefore, for lower percentiles a small change in the percentile (measured by $E_{2}$) can correspond to a large change in volume (measured by $E_{1}$), whereas for higher percentiles the same change in the percentile will correspond to a smaller change in volume. As in Fig. 2, the results in Fig. 3 present results for the 1st and 5th percentiles as columns with different ground truth functions presented in rows, with results for the 10th percentile curves reported in the supplementary material.

**Fig. 4:**
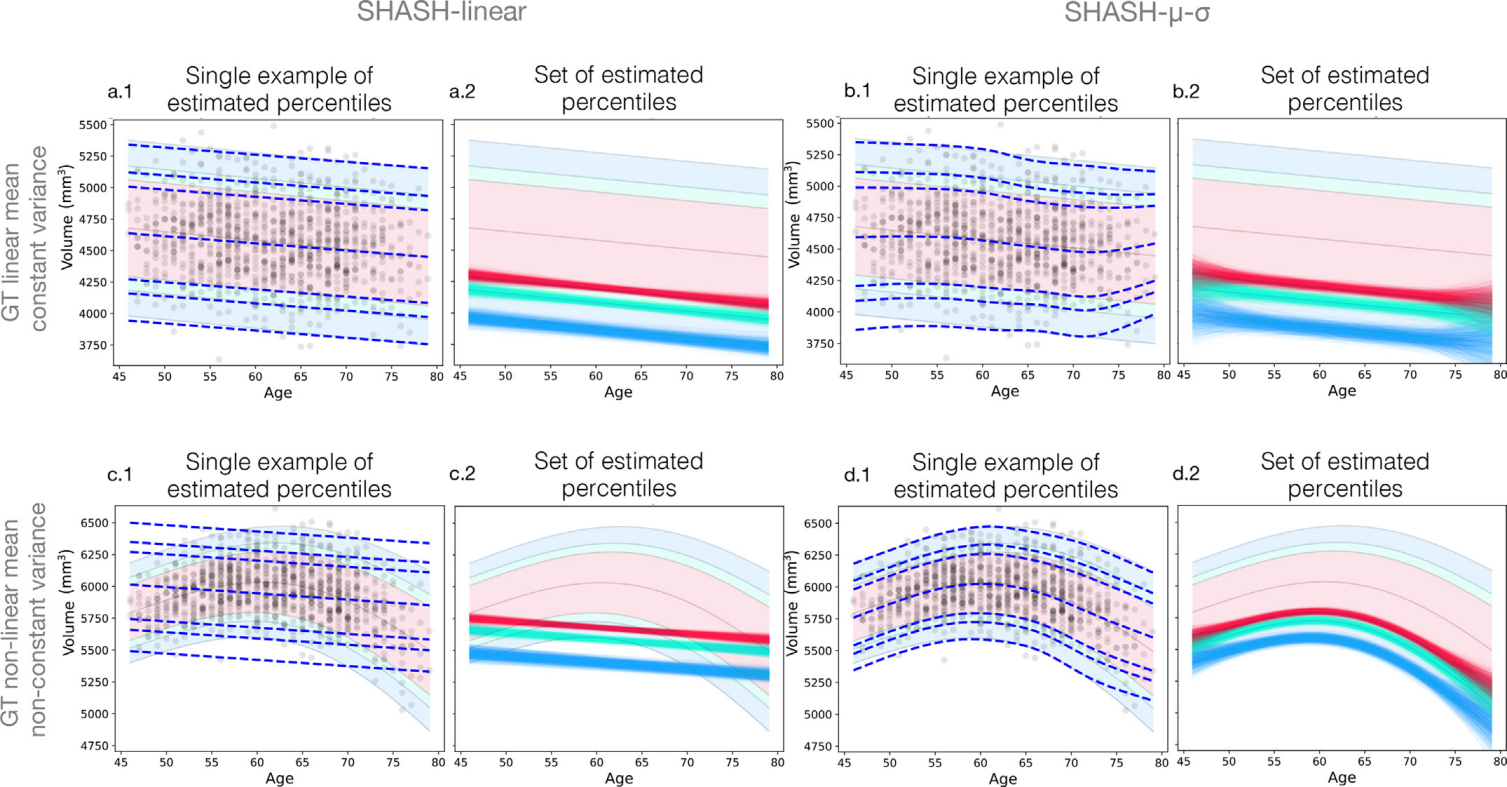
Examples of estimated percentile curves for SHASH-linear (left) and SHASH-μ−σ (right) and ground truth simulation functions (rows) using sample size 1000. Panels are grouped in pairs with the left ones (a1, b1, c1, d1) showing a single example of a simulated sample (black circles) with estimated percentiles (blue dashed lines) overlaid onto the ground truth percentiles of 1, 5, 10, 50, 90, 95 and 99. Panels on the right in each pair (a2, b2, c2, d2) show the 1st (blue), 5th (green) and 10th (red) percentile curves of all simulated samples. (For interpretation of the references to colour in this figure legend, the reader is referred to the web version of this article.)

For all fitting methods and ground truth functions, the errors decrease with increasing sample size, as expected. The MAE of the percentiles, $E_{2}$, also initially decreases with larger sample size, but there is not much improvement at very large sample sizes, especially in the case of the non-linear ground truth (Fig. 3 panels c, d). Non-linear methods show comparable performance to linear methods in the case where the ground truth function is actually linear, but unsurprisingly the linear methods perform poorly in the case of a non-linear ground truth, as indicated by the error $E_{2}$, shown in Fig. 3. However, from Fig. 2 it would appear that the linear methods are outperforming the non-linear methods, which is related to the fact that error $E_{1}$ only assesses the variability in the results, as would be found from any form of stability assessment or bootstrapping type of approach, whereas it is the bias that is the problem in this case, as demonstrated clearly in Fig. 4(c.2). This highlights the fact that both bias and variance should be assessed when evaluating performance of normative models. The differences between individual fitting methods are relatively minor for the most part. However, the sliding window methods nearly always perform weaker than the GAMLSS methods. A closer look at Figs. 2 and 3 indicates that, amongst the GAMLSS variants, the one with the SHASH-μ−σ transformation performs better, for both error measurements, which is most evident when assessing the 5th percentile with non-linear ground truth (panels d in both figures). An apparent discrepancy in the sliding window results in this case can be seen, where they are clearly the worst in Fig. 2(d) but third/fourth best in Fig. 3(d), for high sample size, which can be explained by the fact that large underestimates in volume are typically associated with small changes in percentile errors. In addition, for the non-linear case the sliding window result is better, according to the $E_{2}$ error measure (Fig. 3), than that obtained from some of the other non-linear modelling methods. This is only reliably seen when the sample size is high, which reflects the fact that the sliding window methods are very flexible and therefore they have low inductive biases, although with a tendency to have estimates with higher variance when the sample size is lower. The combination of these factors leads to better $E_{2}$ results for sliding windows than methods with higher biases once the variance no longer dominates. Since these results accord with results from the literature showing superior performance for the GAMLSS method with the SHASH-μ−σ transformation across the methods we tested, we will only show results for this method in further analyses.

### 3.2 Uncertainty across age

The results presented so far show the performance for each fitting method with different sample sizes and methods, but summarising over all ages. Figure 4 illustrates performance as a function of age for both a single example (panels a1, b1, c1 and d1) and summarising over all samples of this size (panels a2, b2, c2 and d2), with a fixed sample size of $N_{s}=1000$ in all cases. The four different pairs of panels show results when varying the ground truth (top and bottom rows for linear and non-linear respectively) or the fitting method (left and right columns for GAMLSS SHASH-linear and GAMLSS SHASH-μ−σ respectively).

As expected, the results are extremely poor for the linear fitting method when applied to the non-linear ground truth (c2). But the linear fitting method is superior, especially at the ends of the age range, when the ground truth is linear (b2 vs. a2). The non-linear fitting method is slightly worse than the linear version when the ground truth is linear, but it nonetheless provides reasonable fits except for the ends of the age range. It provides a vastly better fit for the non-linear ground truth (d2), although again it is less accurate near the ends of the age range. Note that even when the fit is very poor, such as in panel c2, the variance of the estimated percentile curves may be small, even though the bias is very large.

It is important to note that these simulated samples have a smaller number of data points at the ends of the age range. This reflects the properties of many real data sets, e.g. the UK Biobank imaging study, which was used as a basis for our age distribution (see example histogram in Fig. 1). The rapid increase in errors $E_{1}$ or $E_{2}$ at the end of the age range (Figs. 2 and 3) highlights the fact that summary measures over age may hide information that could be extremely important in a number of applications.

**Fig. 5:**
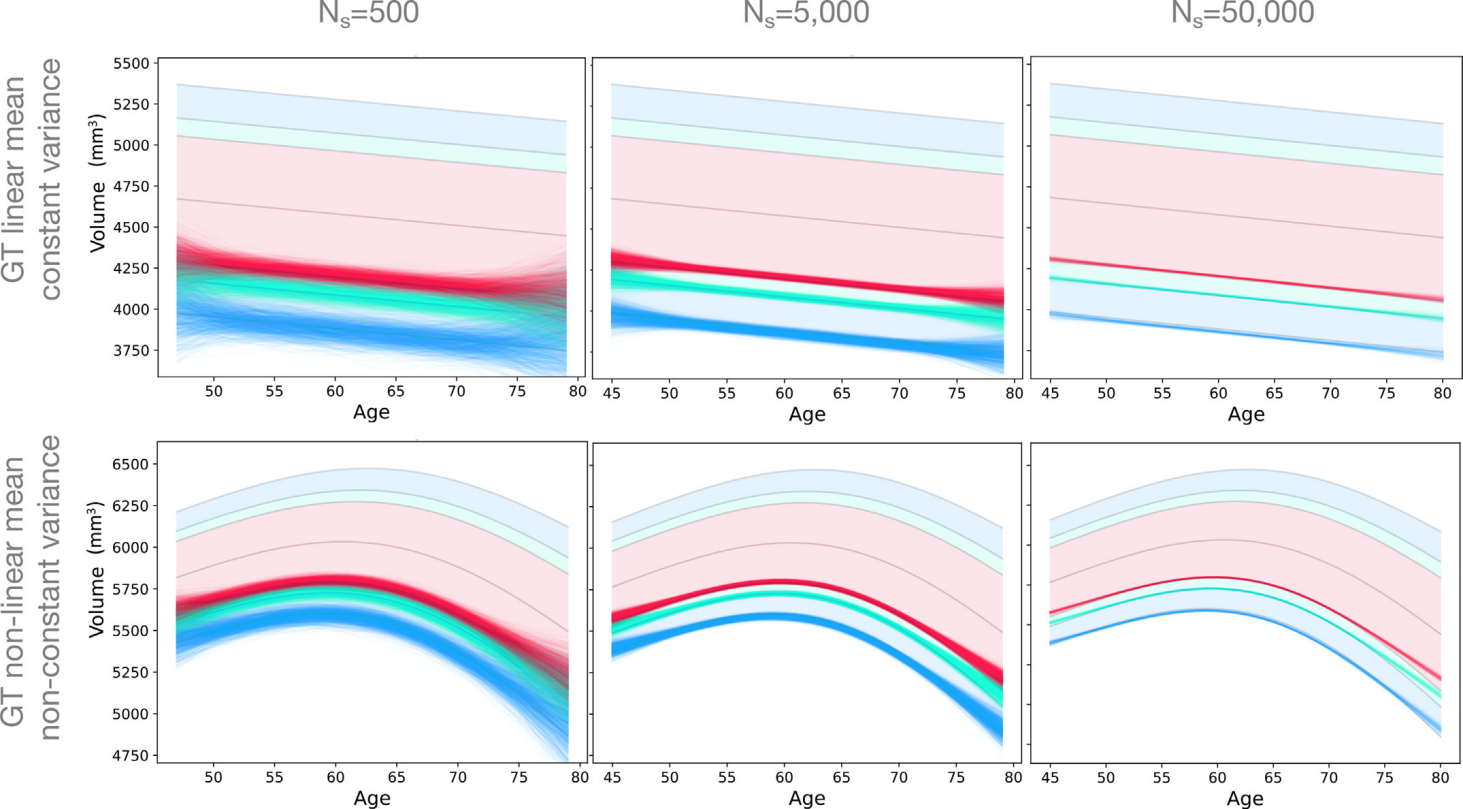
Examples of estimated 1st (blue), 5th (green) and 10th (red) percentile for sample sizes (columns) of 500, 5000 and 50,000 and ground truth simulation functions (rows) using the SHASH-μ−σ model. (For interpretation of the references to colour in this figure legend, the reader is referred to the web version of this article.)

Figure 5 shows how sample size affects the estimated percentile curves across the age range using GAMLSS with SHASH-μ−σ with linear and non-linear ground truths. It can be seen that the variability substantially decreases as the sample size gets larger, although higher variability, as well as bias, can still be observed at the ends of the age range. For example, the 5th percentile curve at the high end of the age range for the non-linear ground truth shows not only variance but noticeable bias. The sample size varies over three orders of magnitude here, and it is only for the largest sample of 50,000 data points that the variability becomes narrow compared to the spacing of the percentile curves.

**Fig. 6:**
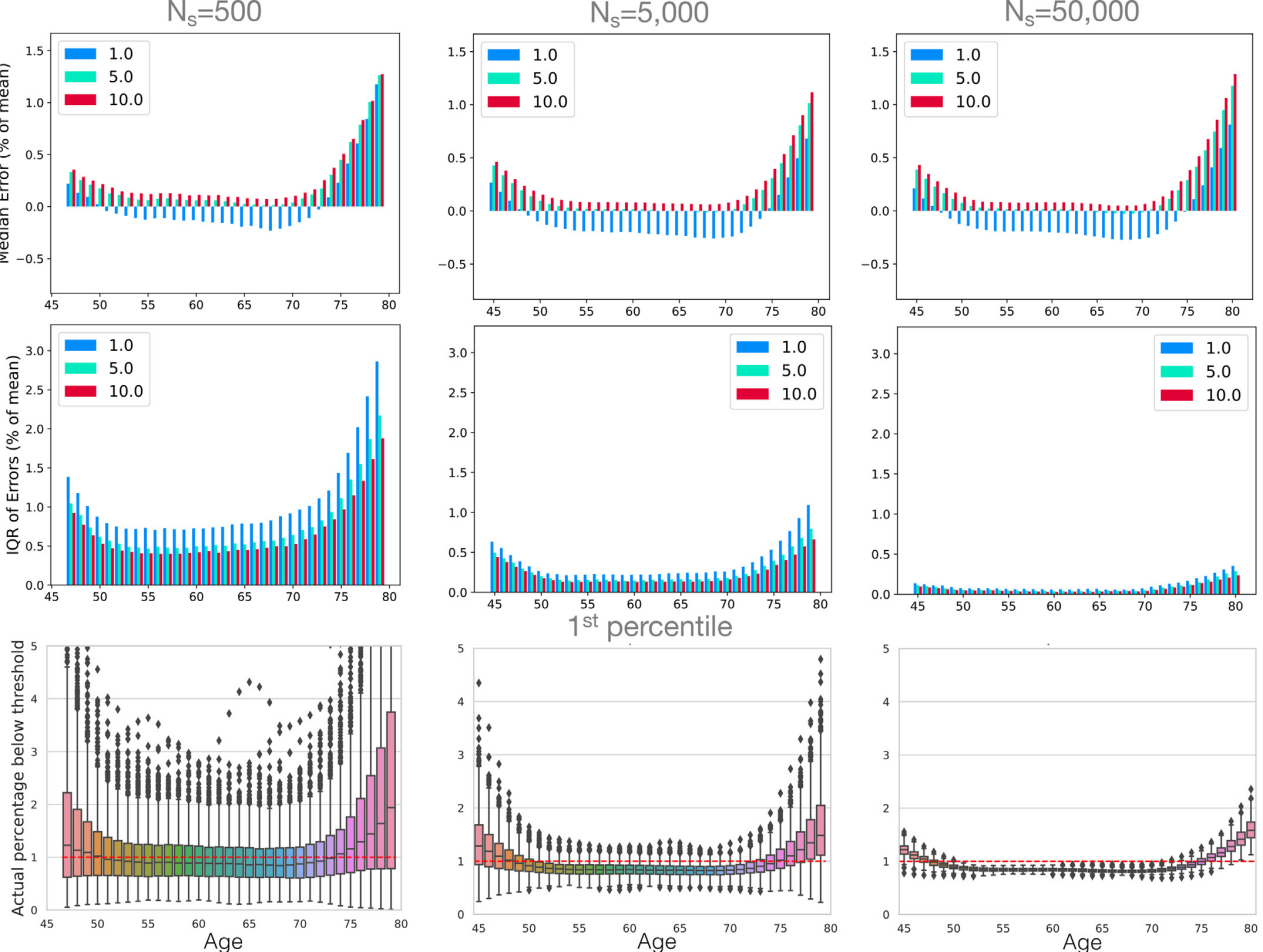
Evaluation of fitting uncertainty with respect to age and sample sizes (columns) 500, 5000 and 50,000 using GAMLSS with SHASH-μ−σ and the non-linear ground truth with non-constant variance. The top row shows the fitting bias, quantified by median error ($E_{1}$), for the 1st (blue), 5th (green) and 10th (red) percentiles. The middle row shows the variance of the fitting, measured by interquartile range IQR of errors ($E_{1}$) as a percentage of the mean. The bottom row shows the actual estimated 1st percentiles and the correct percentile value (red dotted line). Results for the 5th and 10th percentiles are reported in Supplementary Fig. S4 and further sample sizes are reported in Supplementary Fig. S5. (For interpretation of the references to colour in this figure legend, the reader is referred to the web version of this article.)

A more quantitative analysis for performance with respect to age is shown in Fig. 6 for the non-linear ground truth and GAMLSS SHASH-μ−σ fitting method. Here the results are separated into bias quantified by the median error for $E_{1}$ (top row), and variance components quantified by the interquartile range of $E_{1}$ (middle row) for the 1st (blue), 5th (green) and 10th (red) percentiles. These values are expressed as percentages of the mean volume (across all the data), and in these terms the interquartile range in the ground truth is approximately 10% (of the mean volume) and so any errors that are near 10% are roughly equal to the expected IQR from the ground truth, which would represent a very large error. It can be seen from the figure that the bias does not change very much across sample sizes, while variance decreases considerably. With respect to age, both variance and bias are much larger at the ends of the age range, even with large sample sizes, where the values scale with the number of samples of a particular age; for example, the IQR for $N_{s}$ of 5000 at age 80 years is quite similar to the IQR for $N_{s}$ of 500 at age 65 years, as the number of samples are very similar for these (as shown in Supplementary Fig. S1). The same pattern is replicated for each percentile curve, although the 1st percentile is the only one to show noticeable negative bias, associated with the underestimation of the hippocampal volume at the intermediate age groups.

The bottom row in Fig. 6 shows the estimated percentile values using box plots, only for the 1st percentile curve in this case. These values represent the expected percentage of normal participants that would have a value under the estimated percentile curve. The error, $E_{2}$, is equal to the difference between this value and the nominal percentile (p=1). These values have a more intuitive or direct connection to applications where the percentile curves are used to identify individuals as having, or being at risk of, some form of pathology. That is, when estimating the 1st percentile curve we would expect to get $\hat{p}=1\pm Q$%, and it is exactly the quantity $\hat{p}$ that the box plots display, where *Q* (or IQR, a measure of the variation in the results) can be read off the box height (i.e. *Q* is half the height of the box) and the centre of the box plots represents the median value, with any bias shown by deviations of this from the correct value (p=1).

For the case where the sample size is 500 data points (participants), the results are very poor, with the variation (value of *Q*) around 0.5% in the middle of the range and 2–3% at the ends. The whiskers of the box plots are also important to consider, as half of all results will lie outside of the box (and so is closer to a 95% confidence interval). In this case the range of the whiskers is rarely ever below 2% and becomes extremely large at the ends. For 5000 data points, which is larger than that used in a number of normative modelling studies in the neuroimaging literature, the value of *Q* is around 0.2% for much of the age range, but gets close to 1% at the ends of the age range. Furthermore, the range of the whiskers is around 1% in the middle of the age range and reach over 2% at the ends of the range, which could be problematic for some applications (e.g. a cut-off based on the 1st percentile for hippocampal atrophy to support AD diagnosis). Even with a very large sample size (50,000 data points) the size of the whiskers is near 1% at the ends of the age range, though much smaller in the middle where the variation becomes very small and exposes a bias that is larger than the variation. This bias will be strongly related to the inductive bias of the estimation method used, where more flexible models, such as those with more parameters, will tend to have less bias. However, this is often accompanied by a trade-off with increased variance. Box plots for some of the other sample sizes and other percentiles can be found in the supplementary material in Figs. S4 and S5.

## 4 Discussion

Normative modelling has been emerging in neuroimaging in recent years with the availability of big data. From the results shown in this study we want to raise caution when using small or moderate sample sizes for normative data sets. Furthermore, we want to highlight the importance of considering and measuring both variance and bias when evaluating a model, which may not be evident when analysing model performance using a single metric.

We generated samples of different sizes, ranging from 50 to 50,000 data points, consisting of simulated hippocampal volumes for individuals with ages between 45 and 80 years. This age distribution resembled the one in the UK Biobank in order to simulate a real case scenario. We assessed several normative modelling methods, based on sliding window methods or GAMLSS with various settings, using simulated data, with both linear and non-linear functions.

The choice of the hippocampal exemplar used here was done primarily to make the exposition less abstract and more accessible, but should not be interpreted as a restriction on the findings. The considerations should generalise to other curvilinear trajectories (e.g. other subcortical structures in Vinke et al. (2019) and Potvin et al. (2016)), and the approach applied to the lowest percentiles should be equally valid for looking at the 90th, 95th and 99th percentiles for neuroimaging measures where abnormal cases would be represented by higher values (e.g. white matter hyperintensities, as in Fraza et al. (2021)). In fact, making the true distributions slightly simplified, using Gaussian distributions that also have a smaller variability (3-6%) than reported hippocampal values, gives us an optimistic estimate of the performance for normative modelling methods that are applied in situations where the relationships and distributions are broadly similar to those used here. Certain results, such as the absolute numerical values of performance will only apply when the relationships and distributions are similar to those chosen here, but the methodology presented here for estimating and evaluating the performance of normative models is general and applicable to all situations.

The results across all fitting methods that we implemented were generally comparable, although GAMLSS with the SHASH-μ−σ transformation showed slightly better overall performance (Figs. 2 and 3). This was in line with the recent findings and recommendations by Dinga et al. (2021). Our results show that using these more flexible estimation models is beneficial, particularly with larger samples or cases where the ground truth has a non-negligible non-linear component. As expected, it is evident that linear models alone are not good for highly non-linear ground truth distributions, although they demonstrate low variance and hence high repeatability that might make them appear to be working well. Since these linear models do not outperform the more flexible models in the linear ground truth case by very much, we would not recommend the use of purely linear fitting models for normative modelling.

Our results show that a precise estimation of percentiles requires a large sample. Data sets with less than 5000 data points are unlikely to provide accurate estimates for outlying percentiles across the age range (see Fig. 2, Fig. 3, Fig. 5 and 6), even though they are not uncommon in the literature. This is in line with a recent study demonstrating that reproducible brain-wide association studies require thousands of individuals (Marek et al., 2022). Although normative modelling approaches have been suggested to provide increased sensitivity for brain-behaviour associations (Bethlehem et al., 2020), the results suggest that large samples are still needed for this type of analysis. When looking at the different types of error, we observed that for most sample sizes the variance was dominant, whilst for large sample sizes (e.g. $N_{s}=50,000$) the bias tended to dominate, making further increases in sample size less useful in improving performance. This is indicative of the fact that these sample sizes are sufficient to expose the inductive biases, or a priori hypotheses, within the methods, which are normally expressed in terms of a limitation of the functions that can be fit (e.g. limits on the curvature). Increasing the sample size at this point will not reduce the bias appreciably and only changes to the model or estimation procedures (e.g. boosting) can help in reducing the bias further. However, it should be noted that the bias itself was quite small in these examples and so is unlikely to be problematic in practice for distributions that are broadly similar to the ones used here. For other instances, such as during early development, where the relationships and distributions are not similar, the bias could be stronger but the same methodology that is used here can also be applied there to estimate its magnitude.

Many studies and data sets have a relatively small number of participants at the ends of the age range, as in the UK Biobank on which we based our simulated age distribution. The substantial deterioration of the performance at the ends of the age range is likely to be due in large part to this and also in part to the difficulty of constraining flexible models at the end of their range. These issues have also been noted in Dinga et al. (2021); Fraza et al. (2021) where they observed bigger deviations in Gaussian-based models in the tails of the distribution, which contained a relatively small proportion of the data. One way to approach this problem is to have a good sampling strategy, including more participants at the margins of the range or even outside the range of interest (Cole, 2022). This is a scenario where our code can be used to test different sampling strategies to guide recruitment. When using data that is already acquired, merging multiple data sets can improve edge effects. However, besides the challenges related to data harmonisation, some age ranges will still remain difficult to sample, for example the oldest old. A further contributing factor in real data sets is that the ground truth may be more dynamic at the ends of the human lifespan as well as more varied across individuals. Whatever the cause, the uncertainty at either end of the age range means that attempting to go near the ends of the captured age range is likely to yield extremely poor results, especially when the number of samples drops off substantially. Consequently, normative models should not be trusted unless the normative sample is extremely large (Fig. 4, Fig. 5, Fig. 6) and extrapolating beyond the age range should always be avoided, even for very large data sets.

In practice it is usual that only one set of data points is available and the ground truth is unknown, in which case the entire range of possible percentiles shown in the box plots (in Figs. 6, S4 and S5) should be considered, since half of all estimations lie outside the central interquartile range. This would mean that with 5000 participants the estimated 1st percentile curve could be closer to the real percentiles in the range 0.2% to 3%, especially near the ends of the age range. If this was used to screen individuals then it would mean that instead of 1% of normal individuals being labelled as positives (those below the curve) this percentage might actually be as high as 3% (or as low as 0.2%) for those near the ends of the age range for an estimated normative model. The consequence for those participants that actually had a pathology would either be beneficial (a better chance of being detected if the estimated percentile curve was high) or, more problematically, detrimental (less chance of detection if the estimated percentile curve was low). For extreme percentiles, such as the 1st percentile, the low number of samples across the age range can lead to an underestimation of the curvature in the intermediate age range as well. This secondary error can result in reduced detection rates for abnormalities in this intermediate age range. To quantify this in terms of statistical power would require knowledge of the distribution of the main quantity (e.g. hippocampal volume) for participants with pathology, though estimated percentile curves that are lower than the true percentile curve will be likely to lead to high false negative rates.

Recent works have modelled different neuroimaging-derived measures using large data sets providing normative or reference curves across the lifespan (Bethlehem, Seidlitz, White, Vogel, et al., 2022, Rutherford, Fraza, Dinga, Kia, Wolfers, Zabihi, Berthet, Worker, Verdi, Andrews, Han, Bayer, Dazzan, McGuire, Mocking, Schene, Sripada, Tso, Duval, Chang, Penninx, Heitzeg, Burt, Hyde, Amaral, Wu Nordahl, Andreasssen, Westlye, Zahn, Ruhe, Beckmann, Marquand, 2022). Our work complements these by focusing on evaluating performance of commonly used normative modelling methods using simulated samples. Using these we have evaluated the performance across multiple options, showing that summarising over ages can hide poor results and representing the performance as a single metric is likely to be too simplistic. Furthermore, measuring only variance (e.g. using IQR) is not sufficient to judge performance and define an appropriate fitting method, as is illustrated by the performance of the linear models in the case of the non-linear ground truth, since they appear to perform very well according to variance ($E_{1}$) alone, whereas they are clearly performing badly when assessing both bias and variance ($E_{2}$). If only variance would be assessed this will tend to favour simpler models, even though they are likely to include higher inductive biases. Thus, we strongly recommend assessing both bias and variance, because the results showed very different behaviour of these two terms as the sample size is increased, and only through monitoring both can a full picture of performance be gained. However, exact bias measurements can only be made if the ground truth is known, and empirically estimating it reliably requires extremely large samples (as outlined in the methods section) especially since near the end of the age range the number of data points is likely to be smaller. Therefore, using a simulation-based approach can be a very useful method for assessing performance, and for this reason we have made our code available as a general resource for evaluation of normative modelling methods.

The use of these simulation methods can provide estimates of quantitative uncertainties that can be used in statistical testing or reporting of normative results clinically, as suggested in the Introduction. Although the underlying distribution and relationship to the variables of interest cannot be known precisely, a range of similar functions can be simulated, based on an initial exploration of the data set. This exploration should use flexible estimation methods that have low inductive bias (e.g. sliding window) as even though these may not be optimal for estimation of the uncertainties they can give an indication of the form of the relationship that is needed to formulate a suitable range of functions to simulate such that the true function is likely to be captured within the range. We believe that the use of uncertainties obtained in this way would enrich the outputs from normative modelling work and improve statistical practice in the field.

In this work we used age as the only variable included in the model. However, taking into account other variables could be informative. Volumetric data can be corrected for intracranial volume (ICV) to account for head size. In this study, uncorrected data was used as a number of different solutions exist to perform ICV correction (Voevodskaya et al., 2014). However, ICV correction is generally recommended and this simulation-based approach would be applicable to corrected volumes as well. Other variables have been taken into account by generating separate models. For example, different models are usually created for males and females, while recent work generated different models for different genetic risk groups for AD (Ching, Abaryan, Santhalingam, Zhu, Bright, Jahanshad, Thompson, 2020, Janahi, Aksman, Schott, Mokrab, Altmann, 2022, Veldsman, Nobis, Alfaro-Almagro, Manohar, Husain, 2020). The downside of this approach is that the sample size is significantly reduced when the data is split across groups (especially for rare genetic variants). Ideally, the additional variables could be incorporated in the model itself, which is an open direction for future research.

There are several limitations of our work that should be considered. One limitation is the sole use of simulated data. We decided to focus on simulations for several reasons. Firstly, because we wanted to apply and evaluate models on large sample sizes (up to 50,000 data points). With real data this would only be possible by merging data sets from different sites and/or studies (e.g. Bethlehem, Seidlitz, White, Vogel, et al., 2022, Casey, Cannonier, Conley, Cohen, Barch, Heitzeg, Soules, Teslovich, Dellarco, Garavan, Orr, Wager, Banich, Speer, Sutherland, Riedel, Dick, Bjork, Thomas, Chaarani, Mejia, Hagler, Daniela Cornejo, Sicat, Harms, Dosenbach, Rosenberg, Earl, Bartsch, Watts, Polimeni, Kuperman, Fair, Dale, 2018, Thompson, Stein, Medland, et al., 2014). This would then require data harmonisation, which represents a separate issue and active field of research on its own, as the application of normative models and normative ranges in real data sets should be adjusted to effectively deal with site-effects (Kia, Huijsdens, Dinga, Wolfers, Mennes, Andreassen, Westlye, Beckmann, Marquand, Kia, Huijsdens, Rutherford, Dinga, Wolfers, Mennes, Andreassen, Westlye, Beckmann, Marquand).

A second limitation is the use of a single age distribution throughout the work. To make our simulations realistic we used an age distribution based on the UK Biobank, the biggest single-study data set currently available, which has already been used in several normative modelling studies (Janahi, Aksman, Schott, Mokrab, Altmann, 2022, Nobis, Manohar, Smith, Alfaro-Almagro, Jenkinson, Mackay, Husain, 2019). This makes our simulations easier to compare with these studies, with the added advantage of knowing the ground truth distribution to give a sense of how good the estimations are in the real data set studies. It is worth noting that since the UK Biobank age range is restricted to adults, the results achieved with the SHASH implementation of GAMLSS may not be optimal for modelling the early developmental period that has nearly exponential changes and the accompanying increase in participant variability in that age range. Further simulation studies would be required to investigate performance in this early developmental period.

Another limitation is the choice of ground truth distributions, using only two cases: the simplest one of a completely linear function with constant variance and perfectly Gaussian conditional distributions. We did experiment with other distributions, but we believe that these two showed all the interesting effects and spanned a reasonable range of expected true distributions, although still being slightly idealised by the use of Gaussian conditionals. However, by making the ground truth somewhat simpler than might occur in practice and using estimation methods that have a similar but slightly greater flexibility than this, we are creating a situation where the normative models are likely to do slightly better than they might do otherwise. Consequently, we believe that these results are likely to provide optimistic performance estimates.

It would be possible to increase the performance even more in certain cases if less flexible models were used (e.g. fixing the skewness and kurtosis values to match a pure Gaussian distribution, or using a purely linear model for the linear ground truth case) as estimation methods for less flexible models are typically more stable. However, this involves strong assumptions about the nature of the ground truth, which are likely to increase biases that can be hard to detect. Thus, along with Dinga et al. (2021), we would not recommend using models that are quite this restrictive, even though there are benefits with respect to more stable estimations within these normative models. Conversely, increasing the flexibility even more, by allowing the skewness and kurtosis to vary with age, would increase the ability to match a greater range of functions but also greatly amplify the problems in obtaining stable estimates of these parameters as there are strong dependencies between shape and scale parameters that make estimation very difficult and require highly specialised solvers with strong regularisation. Similarly, we have used the default smoothing settings which may not provide optimal results and precise centile estimation, but increasing the flexibility of the smoothing models may result in less precise centile estimation without very heavy regularisation. Therefore, unless there is a good reason to suspect that any changes in distributional shape with age are substantial, it is likely to be safer to avoid the most flexible models. Simulations can assess the stability and reliability of the estimation methods and the impact that different non-Gaussian shapes and smoothing options might have on the key outcomes (e.g. low percentiles). Given that no single method will be optimal in all situations, having the ability to quantitatively assess different options in this way is important.

In general, we believe that the compromise option of the two-parameter SHASH method that we used here (with the additional two parameters being constant, but estimated from the data) with other default settings, is likely to work well in many neuroimaging applications where non-linearity is often mild and the trends are generally slowly varying. Given this choice of method, and the fact that our ground truth has simple Gaussian conditionals, the results we present here are likely to represent optimistic estimates of performance. This, combined with the substantial uncertainties we have observed near the edges of the age range, argues strongly for applying normative modelling only with very large samples.

This approach (code available at https://github.com/jelenabozek/NormativeModelling) can be easily extended to work with different data distributions, and used to evaluate the performance of a normative model even before collecting data. Given a hypothesis on the distribution of the values and the expected/known distribution of data points in the age bins, the simulations can provide an estimate of the accuracy of the percentiles of interest in terms of both variance and bias. This can, in turn, inform power calculations (e.g., how many participants are needed to reach a certain level of accuracy in estimating the 5th percentile for people over 70 years old?) and decisions required for applications in a clinical context (e.g., given a certain normative model, how reliable would a cut-off based on the 5th percentile be for people over 70 years old?).

## 5 Conclusion

Normative modelling of simulated values (e.g. hippocampal volumes) from samples with sizes ranging from 50 to 50,000 confirmed that flexible models perform better (e.g. GAMLSS with SHASH-μ−σ transformation), especially when the ground truth is non-linear. Surprisingly large samples with several thousand data points are needed to accurately capture outlying percentiles across the age range for applications in research and clinical settings. Assessment of the reliability of the model’s estimation of the percentiles is important for the clinical setting and should be carefully considered. Summarising evaluation results into a single summary value would often not be sufficient for assessing performance, especially if it did not include some part that was sensitive to bias, when selecting appropriate fitting methods. Thus, both bias and variance, or something sensitive to both, should be used when assessing the model’s performance. Furthermore, caution is needed when attempting to go near the ends of the age range captured by the source data set, due to the rapid increase in uncertainty at the ends of the age range and, as is a well known general principle, extrapolation beyond the age range should always be avoided. To help with such evaluations of normative models we have made our code available and encourage researchers to use this when developing or utilising normative models.

## Data Availability

The code used for generating the simulated data, fitting the normative models and evaluating models performance is openly available at https://github.com/jelenabozek/NormativeModelling.

## CRediT authorship contribution statement

**Jelena Bozek:** Methodology, Software, Writing – original draft, Writing – review & editing. **Ludovica Griffanti:** Visualization, Writing – original draft, Writing – review & editing. **Stephan Lau:** Writing – original draft, Writing – review & editing. **Mark Jenkinson:** Conceptualization, Methodology, Software, Validation, Visualization, Writing – original draft, Writing – review & editing.

## Declaration of Competing Interest

The authors declare that they have no known competing financial interests or personal relationships that could have appeared to influence the work reported in this paper.

## Data Availability

We have shared the link to our code at the Attach File step.

## Appendix A Supplementary materials

Supplementary Data S1 Supplementary Raw Research Data. This is open data under the CC BY license http://creativecommons.org/licenses/by/4.0/ Supplementary Data S1
