# Supplementary material for: Normative models for neuroimaging markers: Impact of model selection, sample size and evaluation criteria
Source: Neuroimage. 2023 Mar;268:119864. doi: 10.1016/j.neuroimage.2023.119864 (PMC11636675; doi:10.1016/j.neuroimage.2023.119864)
Supplement: Supplementary Data S1 — Supplementary Raw Research Data. This is open data under the CC BY license http://creativecommons.org/licenses/by/4.0/ [file mmc1.pdf]

Appendix A. Supplementary Material

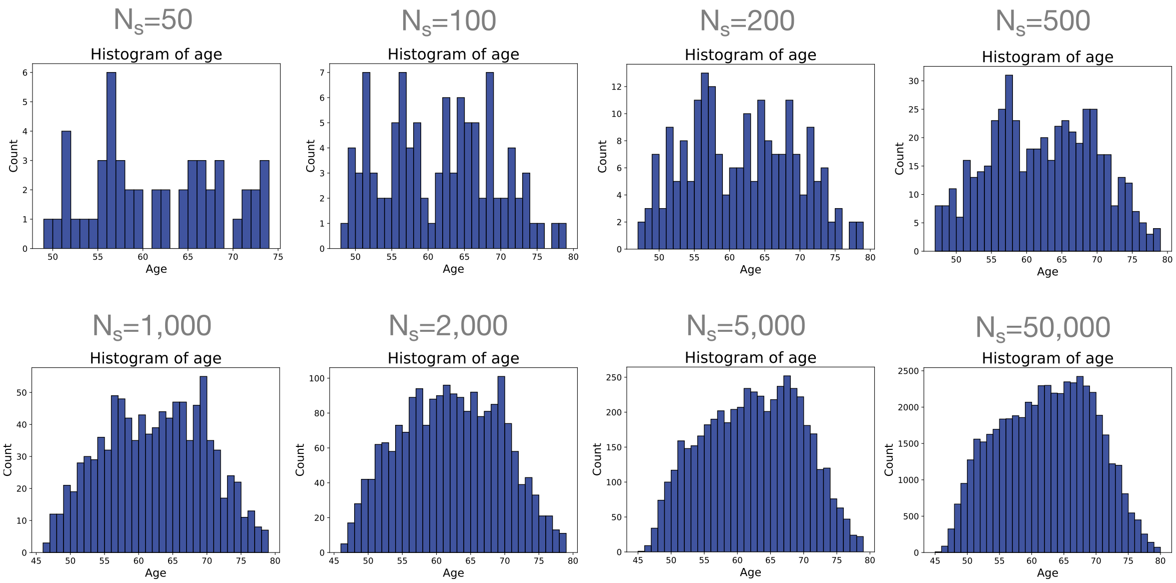

Figure S1: Histograms of ages for the samples used in the simulations. The number of data points is shown in the figure and all simulations with the same number of data points use the same set of ages, hence why there is one fixed histogram for each N_s.

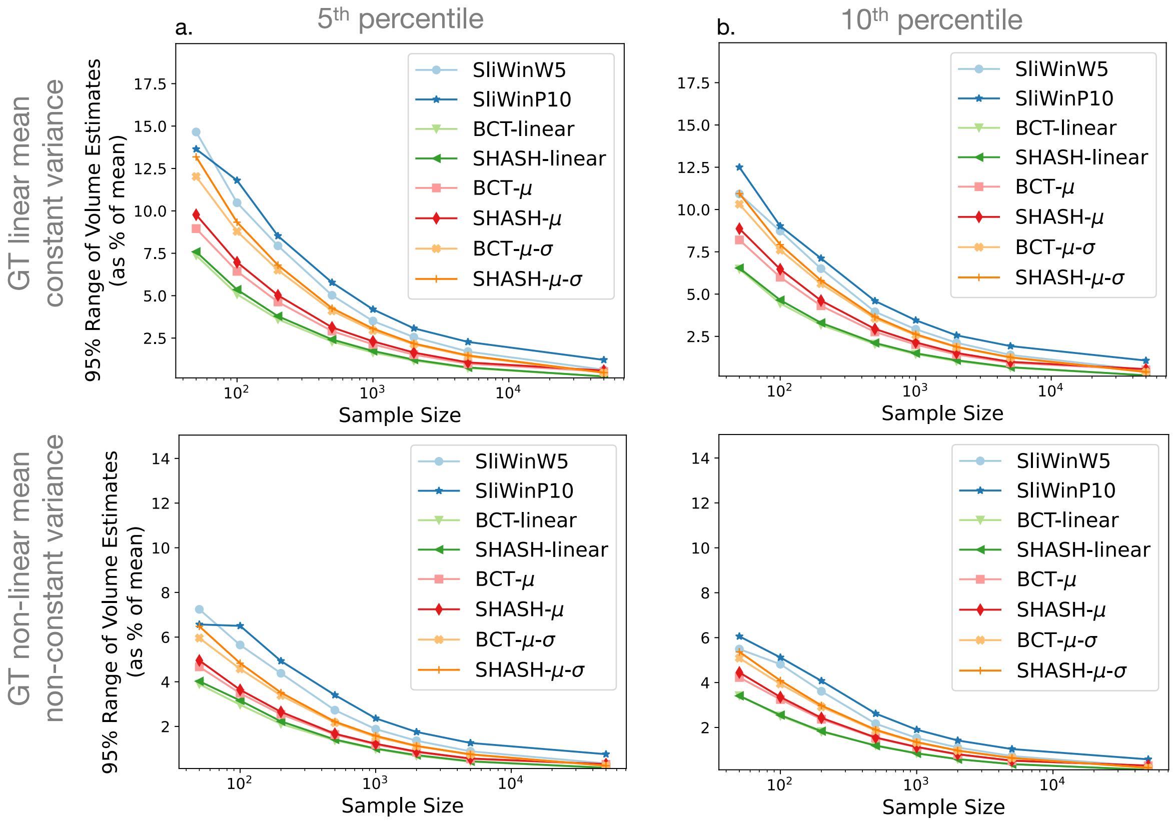

Figure S2: 95% range of volume estimates against sample size for each fitting method, shown as a percentage of the mean. The plots show the confidence intervals (median across age) of the 5th percentile (left column) and 10th percentile (right column) curves for linear mean and constant variance ground truth (top row) and non-linear mean and non-constant variance ground truth (bottom row). Results for the 10th percentile are very similar to those for the 5th percentile (also reported in Figure 2 of the main text).

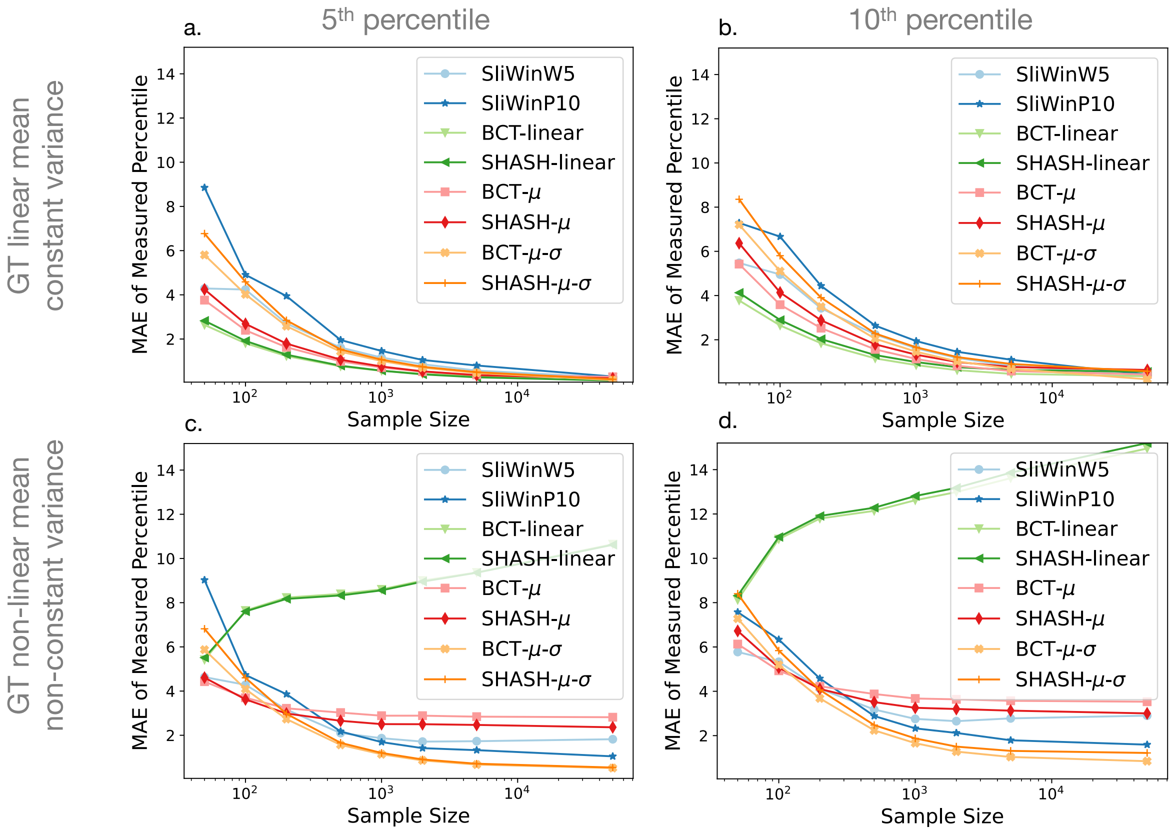

Figure S3: Mean Absolute Error (MAE) against sample size for each fitting method. The plots show the mean across age of the mean absolute error of the estimates of percentile error (E_2) of the 5th percentile (left column) and 10th percentile (right column) curves for linear mean and constant variance ground truth (top row) and non-linear mean and non-constant variance ground truth (bottom row). Results for the 10th percentile are very similar to those for the 5th percentile (also reported in Figure 3 of the main text).

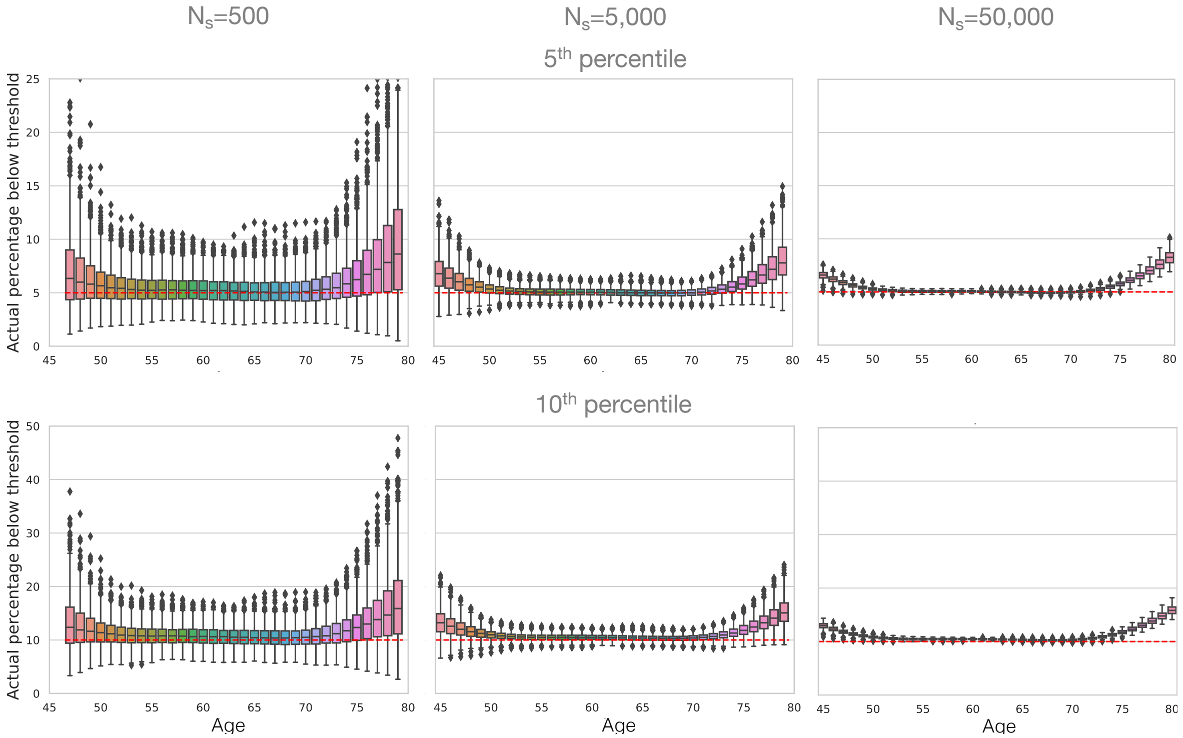

Figure S4: Evaluation of fitting uncertainty with respect to age for sample sizes (columns) 500, 5,000 and 50,000 using GAMLSS with SHASH-μ-σ and the non-linear ground truth with non-constant variance. Actual estimated 5th (top row) or 10th (bottom row) percentiles and the correct percentile value (red dotted line) are shown. Results for 1st percentile are reported in Figure 6 of the main text.

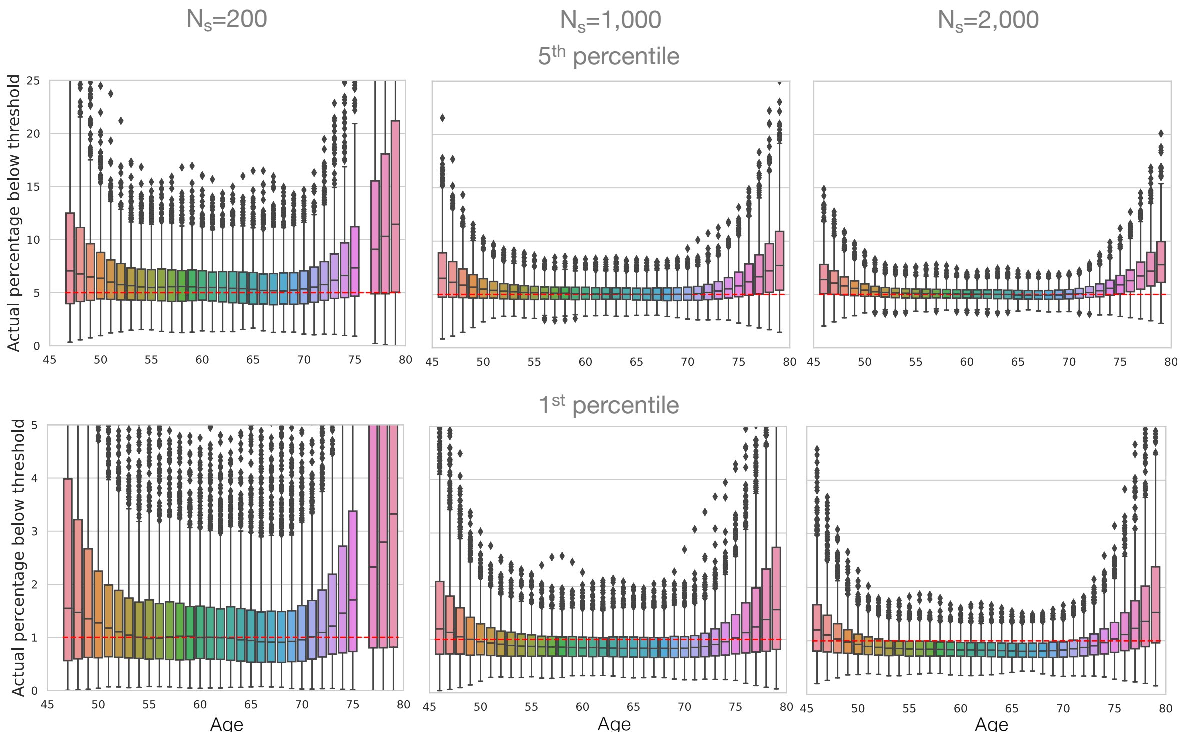

Figure S5: Evaluation of fitting uncertainty with respect to age for sample sizes (columns) 200, 1,000 and 2,000 using GAMLSS with SHASH-μ-σ and the non-linear ground truth with non-constant variance. Actual estimated 5th (top row) or 1st (bottom row) percentiles and the correct percentile value (red dotted line) are shown. Results for other sample sizes are reported in Figure 6 and Figure S4.
